# Case of Abnormal Serous Cyst, Escaping from the Vagina during Parturition

**Published:** 1847-02

**Authors:** 


*Case of Abnormal Serous Cyst, escaping from the Vagina during parturition. Read to the New-York Pathological Society, by* WILLIAM MACNEVIN, M. D., *of New-York.*

Mrs. Murray, aged 30, became pregnant, for the sixth time, before the 1st of February, 1845, and on the 15th of October following, was taken in labor at the full time. On my arrival, about two hours after the commencement of labor, I found the os uteri well dilated, and what appeared to be the distended membranes protuding. I was unable, however, either during the continuance of a pain, or in the interval of repose, to distinguish any presenting part of the child. The pains were very strong, and recurred as often as every two or three minutes. The abdomen of the woman was unusually large and prominent, so much so as to induce her to suppose that she was carrying twins. The os uteri seemed to yield readily to the pains, allowing, as I perceived, the distended membranes to protrude more and more through its orifice. Owing to this circumstance, in twenty minutes after my first examination, on attempting to make a second during the existence of a pain, I found that the membranes protruding through the os uteri had formed a sort of pouch, which so filled up the vagina, that I was obliged to desist from introducing my finger until after the cessation of the pain. The result of this examination was not more satisfactory than the first. I concluded, however, under the circumstances, to await the rupturing of the membranes without interference, in order to allow the process of labor to advance, in the mean time as far as it would. Suddenly, during a severe pain attended with an exclamation from the patient, a large, smooth mass was expelled through the vulva into the bed, passing over my hand in its course. Raising quickly the bed clothes, I discovered a large, smooth, membranous cyst, of an oval form, and about the size of a sheep's bladder; it was distended with a thin fluid of which it could not have contained less than two quarts. Its surface was perfectly smooth except at one place, where there was a loose membranous appendage formed by a fragment similar to, and continuous with the external layer of the membrane of which the cyst appeared to be composed. This undoubtedly was the only point of attachment which it could have had, when in the cavity of the uterus. No liquor amnii accompanied its expulsion, nor did the bulk of the woman's abdomen appear very materially reduced by it; but before I could remove it from the bed, which I immediately attempted to do, without breaking it, a severe pain siezing the woman caused the bursting of the membranes within the uterus, and a sudden discharge of a quantity of liquor amnii, sufficient to deluge the bed. Unfortunately, whether owing to the force of the waters, or to the crowding against it of the woman, the cyst, which I very anxious to remove from the bed uninjured, broke almost at the same moment and mingled its contents with the liquor amnii, which, I might here observe, the former very much resembled. The cyst which I presented at the last meeting, the gentlemen of the Society have had an opportunity of seeing; it was fortunately not so much injured as to prevent a pretty correct opinion from being formed of its original dimensions. On making an examination to ascertain the nature of the presentation, I found that the same accident to the child which had attended the previous confinement of the mother, had very singularly occurred. Several inches of the cord had escaped before the head, and the child, which was a remarkably large one, was in consequence lost before its delivery was effected. Now a question arises as the nature of this phenomenon; was it an enormous hydatid cyst, simply connected with the ovum, or was it a distinct blighted ovum, of which the fœtus had at a very early period been absorbed? I have not been able to consult a sufficient number of authorities, to know whether a case precisely analogous has been recorded in favor of these latter hypotheses. John Burns observes, "hydatids may enlarge the womb; and these frequently are formed in consequence of the destruction of the ovum." After describing the usual form in which they appear, he says, "sometimes there is only one large hytadid, or at most a very few in the womb. In the advanced stage, we find the belly swelled as in pregnancy; but the breasts, although sometimes tense, are oftener flaccid, and no child can be discovered in utero, nor does the woman feel any motion. There may be pain in the abdomen, and obscure fluctuation is discernible externally, whilst per vaginam it is more distinct. The neck of the womb is small, and the case much resembles ovarian dropsy, except that the tumor occupies the region of the uterus. The duration of this complaint is uncertain; but the water is at last discharged suddenly, and after making some exertion. The bag afterwards comes away, and the process is not attended with much pain. This disease, a solitary hytadid, is oftener combined with pregnancy, or with a mole, than met with alone. The first combination is not uncommon, and I have seen the hydatid expelled some weeks before labor." Kirkringius, p. 28, considers dropsy of the uterus as impossible, and says, that every case of collection of water depends upon a large hydatid. Dr. Denman seems to be much of the same opinion. A member of this Society, Dr. Taylor, mentioned to me two or three days ago, a case of abortion occurring in his own practice, in which he obtained an ovum of six weeks having a single hydatid attached to it, of a size exceeding that of the ovum itself. In regard to the specimen forming the subject of this paper, though its large size suggested the possibility of its being a distinct blighted ovum, yet the total absence, on any part of it, of all remains of placenta or cord, as alluded to by Churchill, leaves little or no doubt that it has been a large hydatid, which has been attached to the chorion in a manner happily illustrated in the specimen obtained by Dr. Taylor.—*N. Y. Annalist.*

